# A VirB4 ATPase of the mobile accessory genome orchestrates core genome-encoded features of physiology, metabolism, and virulence of *Pseudomonas aeruginosa* TBCF10839

**DOI:** 10.3389/fcimb.2023.1234420

**Published:** 2023-07-27

**Authors:** Lutz Wiehlmann, Jens Klockgether, Anna-Silke Hammerbacher, Prabhakar Salunkhe, Sonja Horatzek, Antje Munder, Janno Florian Peilert, Erich Gulbins, Leo Eberl, Burkhard Tümmler

**Affiliations:** ^1^ Department of Pediatric Pneumology, Allergology and Neonatology, Hannover Medical School, Hannover, Germany; ^2^ Research Core Unit Genomics, Hannover Medical School, Hannover, Germany; ^3^ Biomedical Research in Endstage and Obstructive Lung Disease, German Center for Lung Research, Hannover, Germany; ^4^ Department of Surgery, University of Cincinnati College of Medicine, Cincinnati, OH, United States; ^5^ Institute of Molecular Biology, University Hospital Essen, University of Duisburg-Essen, Essen, Germany; ^6^ Department of Plant and Microbial Biology, University of Zurich, Zurich, Switzerland

**Keywords:** accessory genome, cystic fibrosis, genomic island, *Pseudomonas aeruginosa*, quorum sensing, signature tagged mutagenesis, virulence

## Abstract

*Pseudomonas aeruginosa* TBCF10839 is a highly virulent strain that can persist and replicate in human neutrophils. Screening of a signature-tagged mutagenesis (STM) TBCF10839 transposon library in phagocytosis tests identified a mutant that carried the transposon in the VirB4 homolog 5PG21 of an integrative and conjugative element (ICE)-associated type IV secretion system of the pKLC102 subtype. 5P21 TBCF10839 insertion mutants were deficient in metabolic versatility, secretion, quorum sensing, and virulence. The mutants were efficiently killed in phagocytosis tests *in vitro* and were avirulent in an acute murine airway infection model *in vivo*. The inactivation of 5PG21 silenced the *rhl*, *las*, and *pqs* operons and the gene expression for the synthesis of hydrogen cyanide, the antimetabolite l-2-amino-4-methoxy-*trans*-3-butenoic acid, and the H2- and H3-type VI secretion systems and their associated effectors. The mutants were impaired in the utilization of carbon sources and stored compounds that are not funneled into intermediary metabolism. This showcase demonstrates that a single gene of the mobile accessory genome can become an essential element to operate the core genome-encoded features of metabolism and virulence.

## Introduction

The metabolically versatile γ-proteobacterium *Pseudomonas aeruginosa* is ubiquitously distributed in aquatic habitats and can colonize inanimate and animate surfaces (Grace et al., 2022; Wilson and Pandey, 2022). Being an opportunistic pathogen, *P. aeruginosa* causes a wide range of syndromes in humans that can vary from local to systemic, subacute to chronic, and superficial and self-limiting to life-threatening (Tümmler, 2022). During the last decades, the inherently multidrug-resistant bacterium has become one of the most frequent causative agents of nosocomial infections associated with substantial morbidity and mortality. Chronic airway infections with *P. aeruginosa* are a major cause of morbidity in people with bronchiectasis, chronic obstructive pulmonary disease (COPD), or cystic fibrosis (CF) (Parkins et al., 2018; Vidaillac and Chotirmall, 2021; Greenwald and Wolfgang, 2022).

The 5.5 to 7 Mbp large genome of a *P. aeruginosa* strain consists of the conserved core genome and the variable accessory genome (Abram et al., 2022). The core genome, with few exceptions of loci subject to diversifying selection, is highly conserved among clonal complexes and shows sequence diversities of 0.5%–0.7% among the two major phylogroups, which make up more than 95% of the current *P. aeruginosa* population (Hilker et al., 2015; Freschi et al., 2019; Botelho et al., 2023). The accessory genome consists of extrachromosomal elements like plasmids and blocks of DNA inserted into the chromosome at various loci (Kung et al., 2010; Abram et al., 2022; Botelho et al., 2023). The elements of the accessory genome called “regions of genomic plasticity” (RGPs) (Mathee et al., 2008) can be present in subgroups of the *P. aeruginosa* population but may also occur only in single strains. The individual composition of the accessory genome accounts for most intra- and interclonal genome diversity in *P. aeruginosa* ((Klockgether et al., 2011; Klockgether et al., 2023).

Most RGPs belong to one of the four major categories, i.e., i) integrative and conjugative elements (ICEs) (Johnson and Grossman, 2015), ii) replacement islands, iii) pro-phages and phage-like elements, and iv) transposons, insertion sequences, and integrons (Kung et al., 2010). ICEs possess both plasmid and phage-associated DNA properties (Burrus et al., 2002). Like plasmids, ICEs can exist as circular extrachromosomal elements and are transferred by self-mediated conjugation. Like phages, ICEs can undergo phage integrase-mediated chromosomal integration via site-specific recombination between an ICE recombination site (*attP*) and a recombination site on the bacterial chromosome (*attB*) (Pradervand et al., 2014). The *P. aeruginosa* ICEs are bipartite genomic islands, which may vary between 80 and 200 kb in size (Klockgether et al., 2011). On the one hand, an ICE encodes a cargo of island-specific genes that endow the host strain with highly specialized features of (secondary) metabolism, motility, virulence, or stress response. On the other hand, *P. aeruginosa* ICEs share a syntenic set of 72 open reading frames (ORFs), the majority of which have homologs in numerous β- and γ-proteobacteria (Kung et al., 2010). This conserved backbone is predicted to confer the mobility of the ICE, i.e., excision, self-transfer to a new host, and reintegration. The co-existence of episomal and chromosomal forms and the spontaneous mobilization and transfer have been demonstrated for a few ICEs such as pKLC102 (Klockgether et al., 2007) and the *clc* element (Pradervand et al., 2014; Carraro et al., 2020). Moreover, as a major step forward, Daveri and colleagues (2023) have recently characterized the conjugation system of the ICE*clc* element of *Pseudomonas putida* UWC1. The 20-kb conserved ICE*clc* conjugative transfer region encodes structurally analogous components to known type IV secretion systems (T4SSs), as well as several crucial components not present in other T4SSs. By in-frame deletion and complementation, 15 genes were found to be essential for ICE transfer (Daveri et al., 2023). Thus, based on the criteria of gene content, order, and homology, the lineage of ICE-associated T4SS (Kung et al., 2010; Daveri et al., 2023) is distinct from the two major canonical classes of T4SS in Gram-negative bacteria, denoted A and B (Christie and Vogel, 2000; Galán and Waksman, 2018; Sheedlo et al., 2022).

The pathogenicity of *P. aeruginosa* can vary from commensal bystanders to highly virulent strains (Tümmler, 2022). For example, the highly virulent TBCF10839 strain can escape killing by neutrophils, the most important antipseudomonal weapon of the human host (Klockgether et al., 2013). Viable TBCF10839 bacteria survive in phagosomes, disrupt the phagosomal membrane, and can grow and divide in the cytoplasm of the neutrophils. To resolve the genetic origin of this uncommon pathogenicity trait of intracellular survival in neutrophils, we generated a signature-tagged mutagenesis (STM) (Hensel, 1998) transposon library of *P. aeruginosa* TBCF10839 (Wiehlmann et al., 2007). When pools of transposon mutants were screened for their survival in neutrophils, the STM scan identified a loss-of-function mutant in which the transposon had disrupted an ORF of the T4SS of the pKLC102-like ICE of TBCF10839. We examined the impact of the loss-of-function mutation on numerous features *in silico*, *in vitro*, and *in vivo* and uncovered a plethora of unexpected bacterial phenotypes highlighting that the inactivation of a single per se dispensable gene of the accessory genome can affect gene expression, metabolism, signaling, fitness, and virulence commonly attributed to all *P. aeruginosa* strains.

## Materials and methods

### Bacterial strains

*P. aeruginosa* TBCF10839 (Tümmler et al., 1991) was isolated from the respiratory secretions of an individual with CF who was regularly attending the CF clinic of Hannover Medical School. Storage of bacterial isolates and documentation of clinical data were performed according to the regulations of the CF biobank approved by the Ethics Committee of Hannover Medical School, study no. 6790. Strain TBCF10839 is susceptible against all commonly used antipseudomonal antimicrobials (broad-spectrum penicillins; second-, third-, and fourth-generation cephalosporins; aminoglycosides; and fluoroquinolones) (Klockgether et al., 2013). First, subcultures were maintained in lysogeny broth (LB) supplemented with 15% (w/v) glycerol at −80°C until use. For GeneChip expression analysis and assays on quorum sensing, bacteria were growing in a mineral ABC medium with 40 mM of citrate as the sole carbon source.

The *P. aeruginosa* TBCF10839 STM transposon library was constructed with the plasposon pTnModOGm (Dennis and Zylstra, 1998) carrying variable signature tags as described previously (Wiehlmann et al., 2002; Wiehlmann et al., 2007). Auxotrophic mutants had been counterselected by the growth of the transposon mutants on a minimal medium with glycerol or benzoate as the single carbon source. *P. aeruginosa* or *Escherichia coli* strains were routinely grown overnight as shaken cultures (230 rpm) in LB at 37°C. LB measuring 5 ml was inoculated with a toothpick of frozen bacterial stock solution and incubated for 16–48 h. Recombinant *E. coli* DH5α strains transfected with pME6010 (Heeb et al., 2000) were grown in the presence of 50 μg/ml of tetracycline, and recombinant *P. aeruginosa* was cultured in the presence of 200 μg/ml of tetracycline.

### Isolation of DNA

*P. aeruginosa* genomic DNA was prepared from cells grown in an LB medium following a protocol optimized for Gram-negative bacteria (Ausubel et al., 1994).

### DNA probe

The genomic probe of *5PG21* gene was prepared by PCR of TBCF10839 DNA (5′-CGCTTGCATGATGTTGTATC and 5′-GAGATGTTCAATCGCAAAGG).

### Genome sequencing

After short-read sequencing on a Genome Analyzer II (Klockgether et al., 2013; Sequence Read Archive (SRA) of the EBI: study Accession No. ERP001300), the TBCF10839 genome was re-analyzed by PacBio SMRT long-read sequencing with an SMRT Cell™ (Pacific Biosciences, Menlo Park, CA, USA) on the *RSII* platform at the Leibniz-Institut DSMZ (Braunschweig, Germany) (Bioproject at NCBI: PRJNA975170; accession No. at NCBI: CP127016).

### Screening of the STM library by phagocytosis assay with granulocytes

Forty-eight *P. aeruginosa* TBCF10839 transposon mutants with preselected signature tags were separately grown in LB at 37°C for 16 h and pooled directly prior to exposure to the polymorphonuclear leukocytes (PMNs) (Bohn et al., 2009). To isolate PMNs, 10 ml of freshly drawn blood (with 100 I.U. heparin) of a healthy donor was mixed with 5 ml of 10% (w/v) hydroxyethyl starch. After most erythrocytes had sedimented (40 min, room temperature), the granulocytes were separated by centrifugation (3,000 × *g*, 15 min) using a lymphocyte separation medium (Lymphoprep, Axis-Shield, Oslo, Norway). The cell pellet was suspended in 1 ml of RPMI1640 and stored on ice. The yield of PMNs was determined by manual counting in a Neubauer chamber. The phagocytosis assay was started by adding 1/10 vol. AB-serum and an aggregate 20-fold excess of the 48 transposon mutants (determined by photometry 0.6 OD_560 nm_ = 10^9^ bacteria/ml).

After incubation for 120 min at 37°C under shaking (200 rpm), the PMNs with the internalized bacteria were separated from the extracellular bacteria by centrifugation (800 × *g*, 10 min), suspension of the cell pellet in 0.3 ml RPMI6140, filtration (nitrocellulose, pore size 2 µm, Sarstedt, Nümbrecht, Germany) and washing with phosphate-buffered saline (PBS), pH 7.4. The filter with the adhered granulocytes was transferred into distilled water and mixed vigorously for 5 min. The bacteria were transferred to new tubes and centrifuged (4,000 × *g*, 10 min), and the pelleted bacteria were plated on LB agar.

After overnight growth, genomic DNA was prepared from the plated bacteria to identify the transposon mutants that had survived within the PMNs. Following the protocol published previously (Bohn et al., 2009), the signature tags were amplified by PCR; the transposon-specific 40-bp sequences were purified from the PCR product by restriction digestion, polyacrylamide gel electrophoresis (PAGE), and gel elution and hybridized on dot blots prepared from all 48 pTnModOGm SigTag donor plasmids. The signal intensity of each dot was compared with that of the corresponding signal of the probe prepared from pooled bacteria grown in parallel on LB agar without *in vivo* selection. Hybridization signals out of the 95% confidence interval of the mean were interpreted to be significantly different from the average signal.

Plasmid rescue was performed to transfer the minitransposon with its flanking sequences as stable episomal plasmids into *E. coli* DH5α. The protocol of Dennis and Zylstra (1998) was modified as follows: 10 µg of *P. aeruginosa* DNA was digested with 40 U of *Pst*I overnight at 37°C in 40 µl of restriction buffer and purified by phenol/chloroform extraction, and the pellet was suspended in 25 µl of TE buffer. An aliquot of 500 ng of restricted genomic DNA was incubated with 1,000 cohesive end ligation units of T4-DNA ligase for 6 h at 16°C in a total volume of 250 µl of ligase buffer. Ligated DNA measuring 40–60 ng was transformed into *E. coli* DH5α, and plasmid-harboring cells were selected with gentamicin (30 µg/ml) on LB agar. The plasmids were used for sequencing the genomic insertion site of the transposon.

### Generation of mutants

Previous screens of the TBCF10839 STM library (Juhas et al., 2004; Rakhimova et al., 2008; Bohn et al., 2009) had applied complementation in *trans* to verify the causative role of the transposon-inactivated gene for the respective phenotype. In the case of the mutant D8A6, which is the topic of this report, heterologous host cells either expelled the episomal recombinant plasmids carrying *5PG21* gene or introduced loss-of-function deletions or frameshift mutations. Thus, to confirm the observed mutation and the disruption of the affected D8A6-ORF as the sole cause of the observed phenotype, additional mutants were generated by targeted allelic replacement by inserting a gentamicin resistance cassette at predefined positions. For this, the sequence was screened for restriction sites as targets for insertions. Two sites were chosen, i.e., a) a *Sma*I site at position 1,812 of the ORF to generate an independent mutant with the same gene disrupted and b) an *Eco*RI site eight positions downstream of the ORF’s stop codon to check for potential polar effects on transcripts encoded downstream of the ORF. PCR products representing genome fragments of 1,200 to 1,500 bp surrounding the target sites were cloned into the vector pBluescript II KS(+) and transformed into *E. coli* host cells for further manipulation. After the insertion of a 754-bp Gm resistance gene cassette into the *Sma*I site or *Eco*RI site, the construct was ligated into the multiple cloning site of vector pEX18T carrying a beta-lactamase resistance and a *sacB* levansucrase gene. After transfer of this construct into strain *P. aeruginosa* TBCF10839 by electroporation, the target cells were grown in M9-benzoate medium supplemented first with gentamicin as positive selection and thereafter as the second step with high concentrations of sucrose as lethal negative selection. Thus, the *P. aeruginosa* bacteria were forced to integrate a Gm-containing fragment of the pEX18T construct into the chromosome by homologous recombination and thereafter to dispose of the other plasmid sequences including *sacB*. The map position and orientation of the Gm cassette and the integrity of the flanking genomic regions were subsequently checked by sequencing in order to confirm the correct position of the Gm cassette and to exclude any secondary mutations eventually introduced during the PCR amplification and cloning steps.

### RNA isolation, electrophoresis, and Northern blotting hybridization

Bacterial cells were harvested by centrifugation at 3,800 × *g* for 2 min at 4°C. Total RNA from approximately 3 × 10^10^ cells was extracted with a modified hot phenol method (Tao et al., 1999). Therefore, bacteria were quickly suspended in 0.5 ml of distilled water and lysed in 7.5 ml of preheated (65°C) phenol-lysis buffer mix (5 ml of phenol [pH 5.5], 2.5 ml of 2% sodium dodecyl sulfate, 30 mM of Na-acetate, and 3 mM of EDTA [pH 5.5]) with vigorous shaking for 10 min. The cell lysate was centrifuged (3,800 × *g*, 20 min), and the supernatant was extracted with 3 ml of phenol-chloroform-isoamyl alcohol (25:24:1, v/v) and then subsequently with 3 ml of chloroform-isoamyl alcohol (24:1, v/v). To pellet the nucleic acids, 0.1 volume of 3 M Na-acetate (pH 5.2) and 2.5 volumes of ethanol were added, and the mixture was incubated at −20°C overnight and centrifuged for 30 min at 3,800 × *g*. The pellet was washed with 5 ml of 70% ethanol and suspended in 175 μl of diethyl pyrocarbonate-treated water. DNA was digested by the addition of 40 U of DNase I and 20 U of SUPERaseIn (Ambion, Cambridgeshire, UK) in DNase I buffer (50 mM of Na-acetate, 10 mM of MgCl_2_, 2 mM of CaCl_2_, pH 6.5) for 30 min at 37°C in a total volume of 200 μl. Then, the RNA was purified with the use of RNeasy columns (QIAGEN, Hilden, Germany) according to the manufacturer’s instructions, and the yield of total cellular RNA was quantified by measuring the light absorption at 260 nm. RNA with a size below 200 bp (e.g., tRNAs and 5S rRNA) is below the cutoff of the column and therefore could not be recovered. All the steps were carried out at 4°C, and RNA was also incubated on ice intermittently during the whole RNA isolation procedure.

RNA samples were separated electrophoretically in 1.2% agarose with 2% formaldehyde as the denaturing reagent, and 1× MOPS buffer (20 mM of 4-morpholinepropanesulfonic acid, 10 mM of Na-acetate, 1 mM of EDTA, pH 7.0) was used as the running buffer. The purity and integrity of RNA preparation were checked with the 16S and 23S ribosomal bands as references. Gel-separated RNA was transferred onto Hybond N^+^ membrane with 20× SSC buffer (3 M of NaCl and 0.3 M of sodium citrate, pH 7.0) for 24 h at room temperature.

For subsequent hybridization, the blot membrane was inserted in a 50-ml plastic tube and incubated with constant shaking for at least 3 h at 42°C with 10 ml/100 cm^2^ prehybridization buffer (50% formamide (v/v), 5× Denhardt’s solution, 5× SSC, and 100 µg/ml of herring sperm DNA). After the addition of the randomly labeled DNA probe, the blot was hybridized for 24 h at 42°C with gentle shaking in a hybridization oven. Subsequently, the membrane was washed first with 6× SSC + 3% (w/v) sodium dodecyl sulfate (SDS) for 5 min at room temperature, then with 2× SSC + 3% (w/v) SDS for 20 min at 42°C, and finally with 0.2× SSC + 3% (w/v) SDS for 20 min at 42°C. Hybridization signals were visualized on the blot by chemiluminescent immunodetection with CDP-Star^®^ as substrate.

### GeneChip microarray analysis

The generation of cDNA and subsequent biotin-ddUTP terminal-labeling steps were performed as described in the manufacturer’s instructions for the *P. aeruginosa* GeneChip (Applied Biosystems, Waltham, MA, USA), using 10 μg of total RNA mixed with random primers (Invitrogen, Waltham, MA, USA) and control *in vitro* transcripts of 10 non-*Pseudomonas* gene sequences. GeneChip hybridization and washing were carried out following the manufacturer’s instructions (Applied Biosystems) and as described previously (Salunkhe et al., 2005).

The *P. aeruginosa* PAO1 GeneChip contains oligonucleotide probes for 5,549 protein-coding genes, 18 tRNA genes, a representative rRNA cluster, and 199 intergenic regions selected from the annotated genome of *P. aeruginosa* strain PAO1 (Stover et al., 2000). In addition, there are probes for 117 genes from *P. aeruginosa* strains other than PAO1 and 14 genes from other species, which can serve as controls. Data analysis was performed using the Affymetrix Microarray Suite software (version 5.0) with Affymetrix default parameters. The average microarray hybridization signal intensity was scaled to 150. Two GeneChips for each strain per condition were compared by the four-comparison survival method (Bakay et al., 2002) to search for genes that significantly changed their signal intensities by the Wilcoxon rank test, with a minimum of a twofold change in all four comparisons. The arithmetic average and the standard deviation of the four comparisons were calculated. As an independent criterion for significantly changed signal intensities, a Bonferroni correction of the signal ratios was applied to account for the number of tests, which in this case was the total number of 5,900 ORFs on the chip. First, the ratio of calibrated and corrected hybridization signals per gene (*S_i_*) obtained from cultures grown under identical conditions was verified to follow a Gaussian distribution, and the variance (σ) was calculated. mRNA transcript levels of a gene (*i*) were considered to be significantly differentially expressed if the ratio *S*(*i*)_A_/*S*(*i*)_B_ or *S*(*i*)_B_/*S*(*i*)_A_ exceeds the threshold (1 + *u*σ), whereby the factor *u* defines that upper boundary of the normalized Gaussian integral Φ(*u*), where Φ(*u*) = *x^n^* matches the Bonferroni-corrected 95% confidence interval in the expression (1 − α) = *x^n^* (here, n = 5,900, α = 0.025, and 0.975 ≪ *x* < 1.0). In summary, changes were only classified as significant if they fulfilled the criteria of the four-comparison survival method and exceeded the threshold of the Bonferroni correction for multiple testing.

### Media for metabolome analysis

Wild-type TBCF10839 and mutant were grown in an M9 medium with 40 mM of glucose.

### Fermentations

For primary cultures, bacteria were directly taken from the glycerol stock into 5 ml of LB in a 10-ml glass tube and incubated for 6 h at 37°C with shaking (180 rpm). Subsequently, the primary culture was inoculated into 250 ml of LB in a 1-L flask and grown at 37°C, 180 rpm to produce “intermediate cultures”.

Fermentations were performed in 1.5 L of medium (+0.025% antifoam) in a 2-L bioreactor (BioFlo 110, New Brunswick Scientific, Edison, NJ, USA). For 3 h prior to inoculation, the medium was maintained at 37°C, agitated with 600 rpm, and streamed with 1.5 L/min pressurized air. After sampling 1 ml to screen on LB agar plates for growth of microbial contaminants, the medium was inoculated with “intermediate culture” to an initial OD_578_ ≈ 0.05. During the subsequent fermentation aeration (1.5 L/min pressurized air), agitation (600 rpm), temperature (37°C), pH, and dissolved oxygen (20% O_2_) were operated by the Primary Control Unit of the BioFlo and continuously documented by the BioCommand software. pH was automatically adjusted to 6.8 ± 0.1 with 0.5 M of H_3_PO_4_ or 1 M of NaOH.

### Extraction of metabolites

A sample equivalent to 20 mg cell dry weight was collected from the fermenter during the mid-exponential and early stationary phase of growth. The bacteria were spun down for 5 min at 4°C. After sampling the supernatant, residual supernatant was removed from the cells by three cycles of suspension in 5 ml of pre-cooled isotonic saline (4°C) and precipitation by centrifugation. The cells were re-suspended in 1.5 ml of pre-cooled (−20°C) methanol containing 12 µg of the internal standard ribitol. Cells were disrupted by two freeze/thawing cycles (15 min in ethanol/dry ice followed by 5 min in water at room temperature). After the addition of 1.5 ml of ddH_2_O and 1 ml of chloroform, the sample was vortexed for 60 s and then centrifuged (13,000 × *g*, 5 min, 4°C). The polar phase measuring 1 ml was transferred to a 2-ml tube, dried under nitrogen flow for 2 to 4 h, and then stored at −80°C for up to 4 weeks.

### Derivatization and GC/MS

Dried samples were dissolved in 40 µl of pyridine containing methoxyamine hydrochloride (20 µg/ml) and incubated at 600 rpm and 30°C for 90 min. After adding 70 µl of *N*-methyl-*N*-trimethylsilyltrifluoroacetamide (Chromatographie Service, Langerwehe, Germany), samples were incubated again (600 rpm, 37°C, 30 min) followed by another 120-min incubation at 20°C. Then, 6 µl of an alkane mix containing decane, dodecane, pentadecane, nonadecane, docosane, octacosane, dotriacontane, and hexatriacontane (2 µg/ml in cyclohexane each) was added in order to allow retention index calculations.

A sample aliquot of 1 µl was injected in a Finnigan Trace gas chromatograph (Thermo Finnigan, San Jose, CA, USA) equipped with a DB-5MS column (J&W Scientific, Folsom, CA, USA). Eluted compounds were analyzed with a trace mass spectrometer (Thermo Finnigan, San Jose, CA, USA) after electron impact ionization. Applied parameters for sample injection, gas chromatography, and mass spectrometry were described before (Strelkov et al., 2004).

### Evaluation of data

The raw gas chromatography mass spectrometry (GC/MS) data were processed with the AMDIS (v.2.1) software of NIST (National Institute of Standards and Technology, Gaithersburg, USA). Thereafter, the metabolites were identified with the Agilent Enhanced ChemStation software (Waldbronn, Germany). Generally, all metabolome analysis experiments were performed with three biological replicates and two technical replicates of each biological replicate. Metabolite concentrations were scaled to the internal standard ribitol and the optical density of cell culture and then calibrated to the total signal intensity of the GC spectra. Metabolite concentrations below the detection limit were set to the lowest measured signal. The median intensity values of the six replicates were applied to statistical analysis. Principal component analysis was performed with the software package PAST 4, version 4.13 (May 2023) (Hammer et al., 2001).

### *N*-Acylhomoserine lactone analysis

For analysis of the *N*-acylhomoserine lactones (AHLs) produced by *P. aeruginosa*, we employed different biosensors in combination with thin-layer chromatography (TLC) (Geisenberger et al., 2000; Steidle et al., 2001). Spent supernatants measuring 250 ml from *P. aeruginosa* cultures grown to an OD_600_ of 1.0 were extracted twice with dichloromethane (250:100 supernatant/dichloromethane). The combined extracts were dried over anhydrous magnesium sulfate, filtered, and evaporated to dryness. Residues were dissolved in 250 μl of ethyl acetate. Samples measuring 10 μl were then applied to C_18_ reversed-phase TLC plates (Merck No. 1.15389) and were separated by using methanol (60% v/v) in water as the solvent. For detection of AHLs, the TLC plate was overlaid with soft agar seeded with the *luxAB*-based AHL biosensor *E. coli* MT102 (pSB403) (detects long-chain and short-chain AHLs; Winson et al., 1998) or the biosensor *Chromobacterium violaceum* CV026 (high sensitivity for short-chain AHLs; McClean et al., 1997). On the basis of the mobilities (*R*_f_-values) of the detected spots, tentative identification of AHLs present in the culture extracts was possible.

### 4-Hydroxy-2-alkylquinoline analysis

*P. aeruginosa* strains were grown in a 50-ml flask in 10 ml of LB with constant shaking at 37°C up to an optical density of OD_578 =_ 2.5. The culture measuring 1 ml was extracted with 2 ml of dichloromethane by vigorous shaking, and the liquid phases were separated by centrifugation at 5,000 × *g* for 10 min. The organic phase measuring 1 ml was dried by evaporation. The pellet was suspended in 50 μl of methanol. Thereof, 8µl was separated on Silica Gel 60 F254 TLC plates (that had been pre-soaked for 30 min in 5% (w/v) aqueous KH_2_PO_4_ solution and then dried for 60 min at 80°C–90°C prior to use) with 5% methanol/95% dichloromethane as the mobile phase. Fluorescent spots were visualized under UV light and photographed.

### Assays of hemolysis and protease secretion

Hemolysis was assessed by the growth of *P. aeruginosa* strains on blood agar plates. Secretion of casein-degrading proteases was examined by growing the analyzed *P. aeruginosa* strains on M9 agar plates supplemented with 0.8% (w/v) casein (Cowell et al., 2003) and (optional) 1–50 mM of a further metabolite. Bacteria were qualified to digest casein when a halo of at least one-third of the diameter of the initial colony emerged. LasA and LasB activities were monitored spectrophotometrically by the lysis of *Staphylococcus aureus* cells (LasA) and by the solubilization of elastin impregnated with Congo red (LasB) as described by Kessler and Safrin (2014).

### Acute murine airway infection model

Infection experiments (Munder et al., 2014; Munder and Tümmler, 2014) were performed on 10 mice per bacterial strain. Survival of groups was compared by Fisher’s exact test. Bacteria were grown in LB overnight at 37°C (230 rpm) to the stationary phase. The bacteria were pelleted by centrifugation (4,000 × *g*, 10 min) and washed twice with sterile PBS, and the optical density of the bacterial suspension was adjusted by spectrophotometry at 578 nm. The intended number of colony-forming unit (CFU) was extrapolated from a standard growth curve, and appropriate dilutions with sterile PBS were made to prepare the inoculum for the mice. To verify the correct dilution, an aliquot was serially diluted on LB agar plates. Ten- to twelve-week-old female mice of the inbred strain C3H/HeN were inoculated with 30 μl of the bacterial suspension via view-controlled intratracheal instillation. This non-invasive application technique via catheter allows controlled delivery of the bacteria to the lungs. During the experiments, mice were maintained in microisolator cages with filter top lids at 21°C ± 2°C, 50% ± 5% humidity, and 12-h light–dark cycle. They were supplied with autoclaved, acidulated water and fed *ad libitum* with autoclaved standard diet. Prior to the start of the experiments, animals were acclimatized for at least 7 days. The weight and rectal temperature of the mice were measured daily, and their body condition was determined using a self-developed score. Murine behavior was scored for the parameter vocalization, piloerection, attitude, locomotion, breathing, curiosity, nasal secretion, grooming, and dehydration. All animal procedures were reviewed and approved by the animal welfare committee of Lower Saxony (“Niedersächsisches Landesamt für Verbraucherschutz und Lebensmittelsicherheit/LAVES”; approval number: 04/787) and performed according to its guidelines.

## Results

### Screening of the TBCF10839 STM library identifies a target in the accessory genome

*P. aeruginosa* TBCF10839 is a highly virulent strain that can persist and replicate in neutrophils, the major antipseudomonal defense in humans (Klockgether et al., 2013). The persistence of bacteria in a professional phagocyte is an optimal bioassay to perform genome-wide scans by STM technology (Hensel et al., 1995). We adapted the protocol to *P. aeruginosa* and constructed an STM minitransposon library in strain TBCF10839 (Wiehlmann et al., 2002; Wiehlmann et al., 2007). When sets of 48 mutants with differential tags were exposed to PMNs in phagocytosis assays *in vivo*, dozens of attenuated clones were identified (Wiehlmann et al., 2007). Of the mutants with consistently high differences in their survival rate in comparison to wild-type TBCF10839, the disrupted gene was present in the PAO1 reference genome except for one mutant in which a gene of the accessory genome was inactivated.

**Figure 1:**
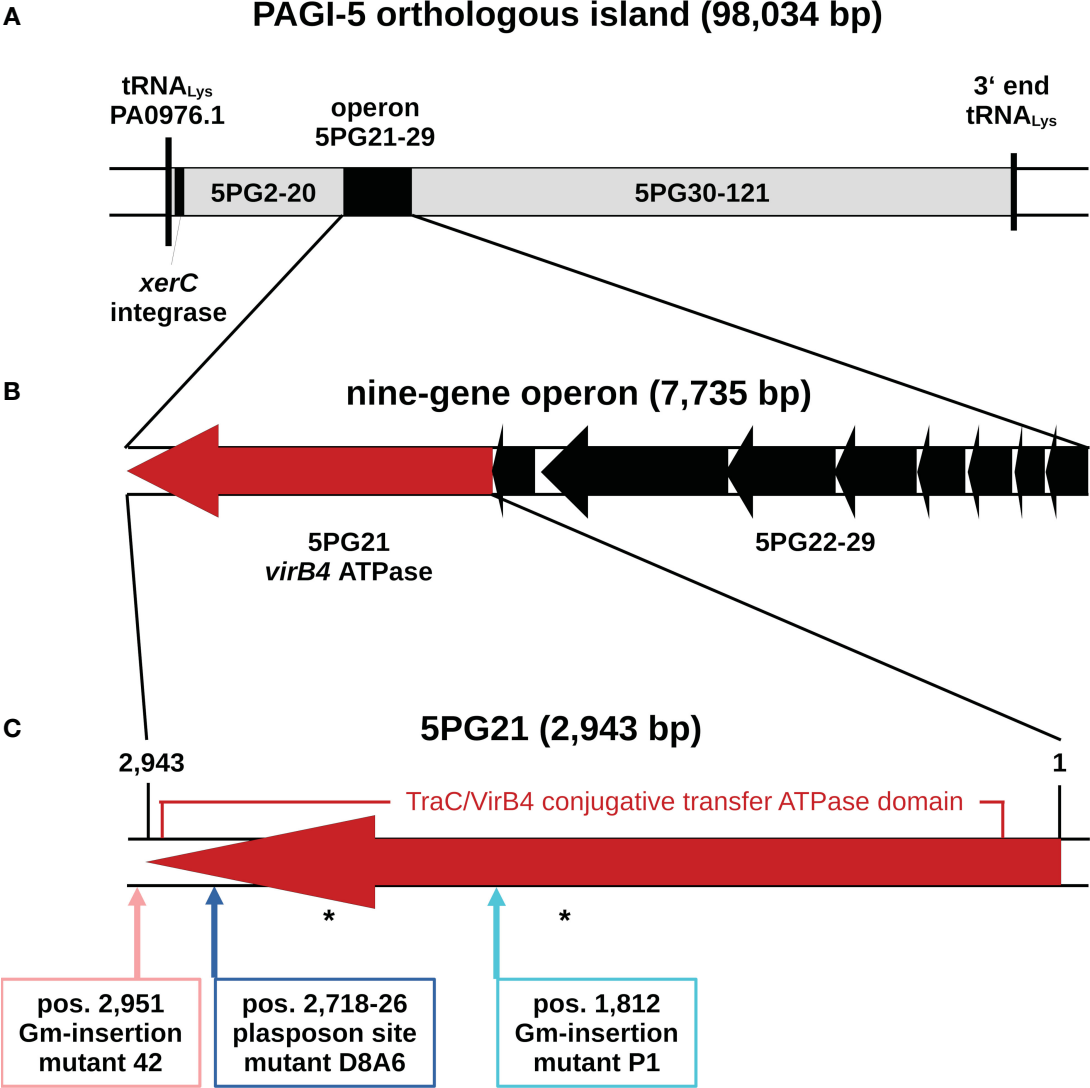
Genomic organization of the pKLC102-type ICE of *Pseudomonas aeruginosa* TBCF10839 that is orthologous to the genomic island PAGI-5 of *P. aeruginosa* strain PSE9 (Battle et al., 2008). **(A)** Map of the ICE in the TBCF10839 genome sequence. The flanking tRNA gene is named in accordance with its counterpart in the PAO1 reference sequence. **(B)** Genetic organization of the nine-gene operon of the ICE conjugative transfer region. Gene orientation is displayed in accordance with the annotation of orthologous island PAGI-5. **(C)** Map of *5PG21* gene homologous to *VirB4*, the only ATPase that is present in all T4SSs. Arrows indicate the positions of the insertions of the plasposon in the D8A6 mutant and of a gentamicin gene cassette in the allele replacement mutants “P1” and “42”. A 2,703-bp segment encoding a TraC/VirB4 conjugative transfer protein family domain is marked. Positions of predicted ATP binding sites are indicated by asterisks. ICE, integrative and conjugative element; T4SSs, type IV secretion systems.

In the case of this mutant, D8A6, the vector fragment and the gentamicin cassette had inserted in the pKLC102-like ICE of TBCF10839 that is orthologous with the genomic island PAGI-5 of *P. aeruginosa* strain PSE9 (Battle et al., 2008) (**Figure 1**). In strain TBCF10839, the plasposon had disrupted a 9-bp segment at positions 2,718–2,726 of a 2,943-bp predicted ORF. The orthologue in strain PSE9 has been named *5PG21*. Since the two genes in PSE9 and TBCF10839 share 100% sequence identity, we also denote the orthologue of TBCF10839 as *5PG21*.

The PAGI-5 ICEs of strains PSE9 and TBCF10839 differ from each other by the insertion of a 1,244-bp large *IS407* element into the intergenic region between ORFs *5PG10* and *5PG11* of PSE9, but otherwise, they share at least 99.98% sequence identity. The *P. aeruginosa* ICEs are split into two subtypes (Kung et al., 2010): ICEs of the pKLC102-subtype such as PAGI-5 that are present in numerous *P. aeruginosa* genomes are endowed with a XerC/XerD-like integrase gene that recognizes a chromosomal *attB* site within the 3′ end of a tRNA^Lys^ gene as an insertion site. Conversely, the ICEs of the *clc* subtype that are widespread in γ- and β-proteobacteria (Klockgether et al., 2011) use their *attB* site to integrate within the 3′ end of tRNA^Gly^ genes, and integration is mediated by bacteriophage P4-like integrase genes (Larbig et al., 2002). Inspection of the TBCF10839 genome revealed homologs of *5PG21* in two *clc*-like ICEs integrated into tRNA^Gly^ genes. The homologs, both of which exhibit approximately 70%–75% sequence identity with *5PG21*, were not identified by the scans of the STM library.

In a current screening of the Pseudomonas Genome Database of 1,071 complete *P. aeruginosa* genomes (Winsor et al., 2016), 426 orthologues of *5PG21* with more than 96% sequence identity were detected, 405 of which showed no gaps in the alignment. Orthologues with 100% sequence identity were identified in 51 genomes. In addition, 692 homologs of the *clc* subtype with 70%–75% sequence identity were detected.

*5PG21* of TBCF10839 is the terminal gene of a nine-gene operon that encodes (part of the) ICE-associated T4SS (**Figure 1**). Taking the criterion of sequence homology, *5PG21* is annotated as the VirB4 ATPase homolog of the “genomic island (GI)-type T4SS” (Kung et al., 2010). Since the VirB4 homolog of the pKLC102 T4SS subtype, but not the VirB4 homolog of the *clc* T4SS subtype (now annotated as *iceB4* (Daveri et al., 2023)), was identified by the scan of the STM library, we hypothesized that features specifically for the pKLC102-subtype could account for the capacity of 5PG21 to promote virulence to TBCF10839. Thus, we next examined the role of 5PG21 in the resistance of TBCF10839 to killing by PMNs.

### PMN phagocytosis assays

**Figure 2:**
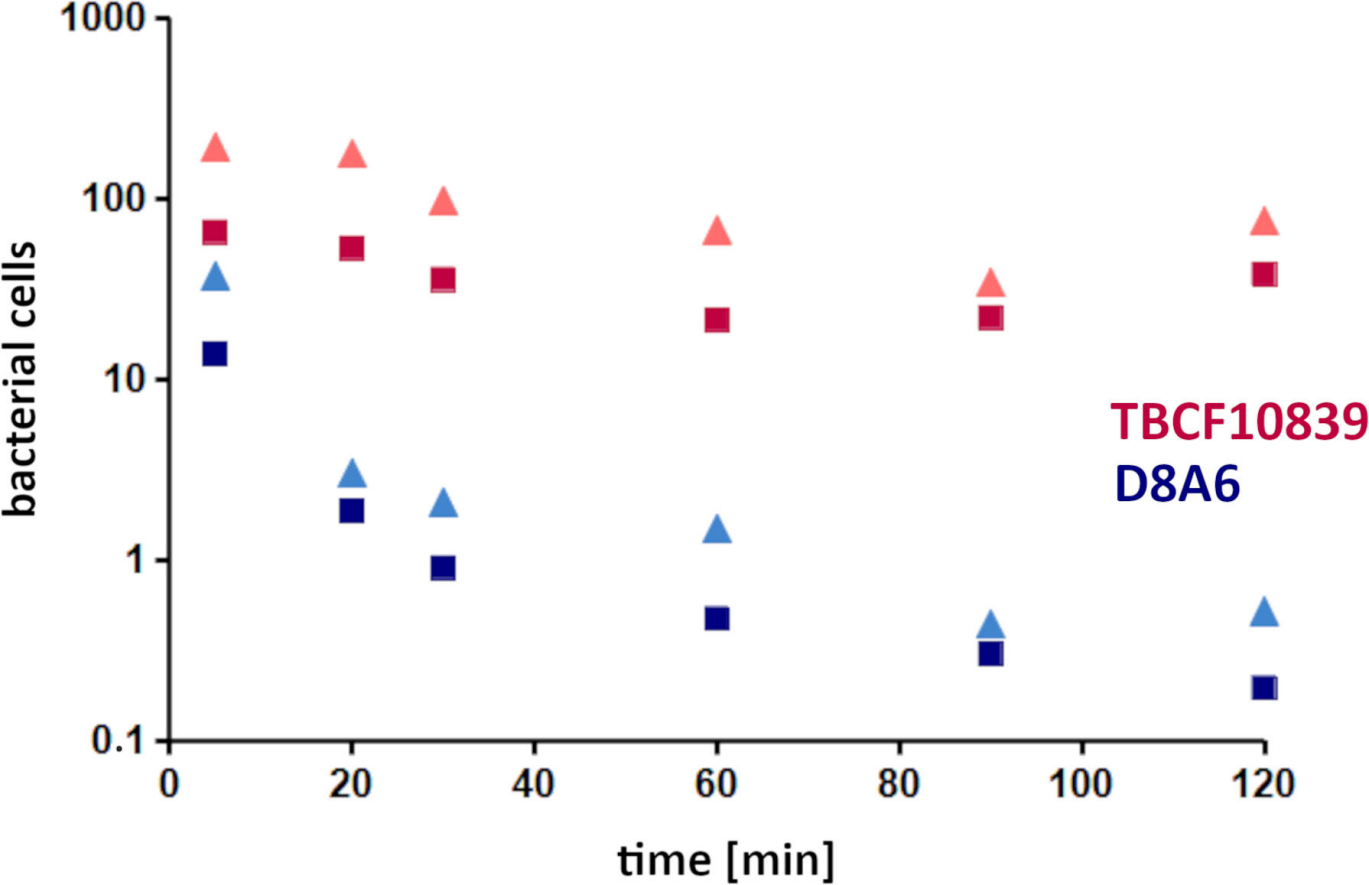
PMN phagocytosis assays with *Pseudomonas aeruginosa* TBCF10839 and its D8A6 plasposon mutant grown to exponential phase (ordinate, 10^6^ CFU/ml; abscissa, time [min]). Phagocytosis assays were performed in parallel with 2.2 × 10^8^ CFU/ml each of wild-type strain and plasposon mutant at an MOI of 10 with PMNs freshly prepared from four independent healthy human donors on separate occasions. Samples were taken at time points 5, 20, 30, 60, 90, and 120 min after exposure of the activated PMNs to the exponentially growing bacteria. Aliquots of 0.0001 of CFU of the assay were plated: red triangle, all TBCF10839; dark red square, extracellular TBCF10839; blue triangle, all D8A6; dark blue square, extracellular D8A6. Symbols indicate the mean of four independent biological replicates. Taking the CFUs of the individual tests, the abundance of total and intracellular bacteria was significantly different between TBCF10839 and D8A6 at all time points (t-test, range of *p-*values: 2 ×10^−6^ to 1 ×10^−4^). PMN, polymorphonuclear leukocyte; CFU, colony-forming unit; MOI, multiplicity of infection.

Mutant D8A6 had been identified during the screening of the STM library when each time a batch of 48 mutants was exposed simultaneously to neutrophils. To verify that mutant D8A6 compared to the wild-type strain is attenuated in its resistance to killing by PMNs, the TBCF10839 parent and its D8A6 mutant were exposed in parallel at a multiplicity of infection (MOI) of 10 to PMNs freshly prepared on separate occasions from four unrelated healthy donors. **Figure 2** summarizes the outcome of the significantly different time courses of viable TBCF10839 and D8A6 bacteria during the 2-h phagocytosis assays (t-test, *p* < 1 ×10^−4^). In the case of TBCF10839, approximately 80% of the inoculum was killed by the neutrophils within the first hour, but then, the number of viable bacteria increased in both the intra- and extracellular compartments. In contrast, close to 90% of the inoculated D8A6 bacteria were immediately killed within the first 5 min, and by 2 h, only 0.1% of bacteria had survived. Correspondingly, the ratio of viable TBCF10839 to D8A6 bacteria increased from approximately 5-fold by 5 min to 50-fold by 30 min and to approximately 200-fold by 2 h. Thus, the phagocytosis assays with singular strains confirmed that compared to its parental strain, the plasposon mutant D8A6 was strongly compromised in its resistance to PMN-mediated killing.

### Gene expression of 5PG21 in TBCF10839 and D8A6

**Figure 3:**
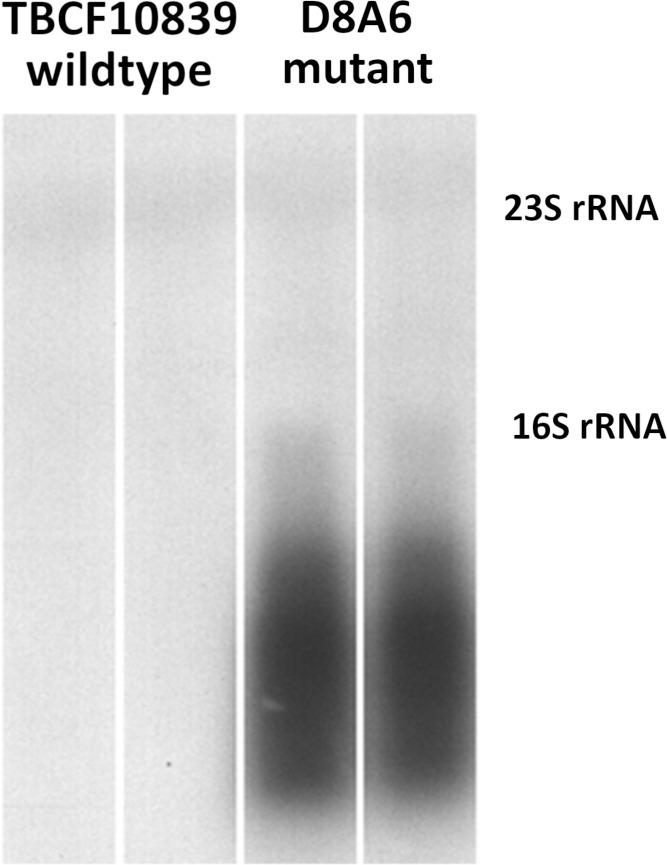
Northern blotting of 5PG21-probe reactive mRNA transcripts in wild-type TBCF10839 and the D8A6 mutant. No specific signal is visible for the wild-type strain, but strong probe-reactive signals of truncated 5PG21 mRNA transcript are seen for the insertion loss-of-function mutant. The faint bands indicate probe-reactive cross-hybridization signals with 23S rRNA (2,890 nt) and 16S rRNA (1,536 nt).

Next, we compared the 5PG21 mRNA transcript levels by hybridization of Northern blots of gel-separated RNA with a genomic probe of *5PG21* gene (**Figure 3**). No probe-positive signal above the threshold could be discerned from the TBCF10839 strain grown in ABC medium with citrate as a single carbon source, but the blot of gel-separated RNA of mutant D8A6 showed a strong and broad probe-reactive signal covering the range from oligonucleotides to the size of 16S rRNA of 1,536 bases. Hence, intact *5PG21* gene was weakly expressed, but the *P. aeruginosa* cell produced large amounts of truncated 5PG21_D8A6_ mRNA transcripts. Since *5PG21* gene is disrupted by the Gm gene cassette, we conclude that the insertion caused excessive production of a non-functional transcript that was subsequently degraded.

### GeneChip comparison of TBCF10839 and the D8A6 mutant

The Northern blotting data indicated that the TBCF10839 strain needs the expression of the yet uncharacterized *5PG21* gene. To obtain some clue about the potential role of 5PG21 in the bacterial cell, we compared the global expression of the core genome of the D8A6 mutant and its TBCF10839 parent. Wild-type and mutant were cultured aerobically side by side in ABC minimal medium in two independent biological replicates. Total RNA was extracted and hybridized onto *P. aeruginosa* PAO1 GeneChips.

Compared to its wild-type parent, the D8A6 mutant significantly upregulated the expression of 122 genes of the core genome (**Supplementary Material**, **Table S1**). The members of the arginine deiminase pathway and the nitrite reductase operon belonged to the 13 mRNA transcripts that were upregulated more than sixfold (**Table 1**). Correspondingly, the intermediates of the urea cycle were present at 1.5- to 2-fold higher levels in D8A6 than in TBCF10839 cells (**Supplementary Material**, **Table S2**).

**Table 1:** PAO1 GeneChip expression analysis of the *Pseudomonas aeruginosa* core genome of TBCF10839 and its isogenic plasposon mutant D8A6: genes differentially expressed by more than sixfold*.

Locus	Annotation	Upregulation in D8A6 mutant compared to TBCF10839
PA0512	nirH	17.9
PA0515	nirD, probable transcriptional regulator	20.1
PA0516	nirF, heme d1 biosynthesis protein NirF	15.3
PA0517	nirC, probable c-type cytochrome precursor	11.4
PA0518	nirM, cytochrome c-551 precursor	6.4
PA0561	Hypothetical protein	6.5
PA2399	pvdD, pyoverdine synthetase D	8.0
PA2566	Conserved hypothetical protein	11.1
PA3153	wzx, O-antigen translocase	8.8
PA4861	Probable ATP-binding component of ABC transporter	11.3
PA5171	arcA, arginine deiminase	9.4
PA5172	arcB, ornithine carbamoyltransferase, catabolic	9.2
PA5173	arcC, carbamate kinase	10.5
Locus	Annotation	Fold-downregulation in D8A6 mutant compared to TBCF10839
PA0111	Hypothetical protein	6.6
PA0122	Aegerolysin rahU, toxin	19.3
PA0263	hcpC, secreted protein Hcp, part of T6SS	45.2
PA0355	pfpI, intracellular protease PfpI	7.7
PA0587	Conserved hypothetical protein	12.7
PA0630	Hypothetical protein	8.5
PA0692	pdtB, phosphate depletion regulated two-partner secretion partner B, transporter PdtB	6.4
PA0852	cpbD, chitin-binding protein CbpD precursor	14.9
PA0997	pqsB, catalyzes the condensation of octanoyl-coenzyme A and 2-aminobenzoylacetate	87.3
PA1002	phnB, anthranilate synthase component II	113.1
PA1003	mvfR, (syn. pqsR), transcriptional regulator	8.8
PA1041	Probable outer membrane protein	10.6
PA1176	napF, ferredoxin protein NapF	13.6
PA1245	aprX, alkaline metalloprotease AprX	13.3
PA1250	aprI, alkaline proteinase inhibitor AprI	9.3
PA1344	Probable short-chain dehydrogenase	18.3
PA1392	Hypothetical protein	7.1
PA1431	rsaL, regulatory quorum-sensing repressor protein RsaL	665.3
PA1432	lasI, autoinducer synthesis protein LasI	8.4
PA1436	Probable RND efflux transporter	11.4
PA1511	vgrG2a, part of H2-T6SS (type VI secretion system, T6SS)	6.3
PA1651	Probable transporter	8.4
PA1656	hsiA2 (part of H2-T6SS)	29.8
PA1658	hsiC2 (part of H2-T6SS)	28.6
PA1659	hsiF2 (part of H2-T6SS)	12.0
PA1660	hsiG2 (part of H2-T6SS)	7.5
PA1661	hsiH2 (part of H2-T6SS)	7.5
PA1664	orfX (part of H2-T6SS)	27.8
PA1665	fha2 (part of H2-T6SS)	121.6
PA1666	lip2 (part of H2-T6SS)	17.4
PA1667	hsiJ2 (part of H2-T6SS)	125.3
PA1669	icmF2 (part of H2-T6SS)	9.3
PA1670	stp1, serine/threonine phosphoprotein phosphatase Stp1	13.6
PA1671	stk1, serine-threonine kinase Stk1	29.3
PA1738	Probable transcriptional regulator	8.0
PA1739	Probable oxidoreductase	8.0
PA1869	Probable acyl carrier protein	60.8
PA1876	Probable ATP-binding/permease fusion ABC transporter	8.8
PA1881	Probable oxidoreductase	6.8
PA1891	Hypothetical protein	8.3
PA1892	Hypothetical protein	10.6
PA1894	Hypothetical protein	14.5
PA1897	Hypothetical protein	22.6
PA1901	Phenazine biosynthesis protein PhzC2	115.4
PA1902	Phenazine biosynthesis protein PhzD2	17.0
PA1903	Phenazine biosynthesis protein PhzE2	7.9
PA1951	fapF, amyloid outer membrane transporter FabF	27.2
PA2068	Probable MFS transporter	11.3
PA2144	glgP, glycogen phosphorylase	8.5
PA2193	hcnA, hydrogen cyanide synthase HcnA	31.9
PA2194	hcnB, hydrogen cyanide synthase HcnB	24.2
PA2195	hcnC, hydrogen cyanide synthase HcnC	14.4
PA2213	Probable porin	7.1
PA2214	Putative l-lyxonate transporter	8.2
PA2305	ambB, non-ribosomal peptide synthetase for l-2-amino-4-methoxy-*trans*-3-butenoic acid (AMB)	9.5
PA2306	ambA, LysE-type transporter for AMB	13.4
PA2365	hsiB3 (H3-T6SS)	6.2
PA2366	hsiC3 (H3-T6SS)	8.0
PA2367	hcp3 (H3-T6SS)	20.7
PA2375	Hypothetical protein	10.6
PA2441	Hypothetical protein	6.3
PA2572	Probable two-component response regulator	28.2
PA2587	Probable FAD-dependent monooxygenase	31.7
PA2592	Probable periplasmic spermidine/putrescine-binding protein	14.3
PA2593	qteE, quorum threshold expression element, QteE	34.6
PA2717	cpo, chloroperoxidase precursor	6.4
PA2746	Hypothetical protein	6.2
PA2747	Hypothetical protein	6.6
PA2927	plaB, phospholipase B	19.0
PA3311	nbda, c-di-GMP-specific phosphodiesterase NbdA	6.6
PA3326	clpP2, ClpP protease positively regulating alginate overexpression	26.8
PA3327	Probable non-ribosomal peptide synthetase	15.5
PA3330	Probable short-chain dehydrogenase	69.6
PA3331	Cytochrome P450	58.6
PA3333	fabH2, 3-oxoacyl-[acyl-carrier-protein] synthase III	20.7
PA3334	acp3, acyl carrier protein 3 Acp3	77.9
PA3476	rhlL, autoinducer synthesis protein RhlL	10.7
PA3477	rhlR, transcriptional regulator RhlR	12.3
PA3478	rhlB, rhamnosyltransferase chain B	11.7
PA3479	rhlA, rhamnosyltransferase chain A	29.7
PA3519	Hypothetical protein	8.2
PA3535	Probable serine protease	9.1
PA3550	algF, alginate *O*-acetyltransferase AlgF	14.4
PA3690	Probable metal-transporting P-type ATPase	7.0
PA3724	lasB, elastase LasB	16.6
PA3906	Co-chaperone, co-TecT (involved in T6SS mediated effector secretion)	131.3
PA3907	tseT, TOX-REase-5 domain-containing effector, TseT (part of T6SS)	29.5
PA3908	tsiT, immunity protein, TsiT (part of T6SS)	173.7
PA3928	Hypothetical protein	7.5
PA4129	Hypothetical protein	16.6
PA4130	nirA, ferredoxin-dependent nitrite reductase, NirA	24.2
PA4132	mpaR, MvfR-mediated PQS, and anthranilate regulator MpaR	8.1
PA4133	Cytochrome c oxidase subunit (cbb3-type)	40.6
PA4134	Hypothetical protein	35.3
PA4139	Hypothetical protein	138.4
PA4141	Hypothetical protein	61.3
PA4142	Probable secretion protein	18.5
PA4143	cvaB (syn cyaB), probable toxin transporter	8.1
PA4190	Probable FAD-dependent monooxygenase	6.5
PA4209	phzM, probable phenazine-specific methyltransferase	37.6
PA4738	Conserved hypothetical protein	14.8
PA4739	Conserved hypothetical protein	21.4
PA4778	cueR, negative regulator of H2-T6SS dependent copper binding, regulator of surfing motility, CueR	7.0
PA4828	Conserved hypothetical protein	10.3
PA4876	osmE, osmotically inducible lipoprotein OsmE	6.3
PA4925	Conserved hypothetical protein	10.8
PA5220	Hypothetical protein	11.0
PA5401	Hypothetical protein	6.7
PA5446	Hypothetical protein	10.3
PA5460	Hypothetical protein	8.9
PA5482	Hypothetical protein	16.4

*Strains were grown side by side in ABC medium with 40 mM of citrate as a single carbon source up to the late exponential phase (optical density at 600 nm of 2.7 to 3.0). The table lists all mRNA transcripts that were significantly differentially expressed after Bonferroni correction for multiple testing (see Material and methods). Genes were annotated as described in the original publications and/or the Pseudomonas Genome Database (accessed 10 May 2023).

Wild-type TBCF10839 significantly expressed 234 genes of the core genome more strongly than the D8A6 mutant (**Supplementary Material**, **Table S2**), 111 and 70 of which by more than 6- and 10-fold, respectively (**Table 1**). Apart from several yet uncharacterized hypotheticals, only a few upregulated gene products were involved in metabolism. Instead, the majority of differentially expressed transcripts are relevant for transport, secretion, signaling, and virulence. Major functions are the synthesis of the O-antigen, hydrogen cyanide, the antimetabolite l-2-amino-4-methoxy-*trans*-3-butenoic acid, and the production of type VI secretion systems (T6SS) and their associated effectors such as the H2-T6SS cluster, the TseT-TsiT effector–immunity protein pair, and the anterior part of the H3-T6SS virulence locus involved in biofilm formation (PA2364-PA2369). On top of this, the *rhl*, *las*, and *pqs* operons of quorum sensing (QS) were consistently more strongly expressed in TBCF10839 than in its D8A6 mutant (median 12-fold; range 5.6- to 660-fold).

### Construction of further mutants by allelic replacement

The microarray data indicate that the transcriptome of the D8A6 mutant is compromised in numerous features that *prima facie* one would not ascribe to a loss-of-function mutation in a VirB4 homolog of a T4SS (Daveri et al., 2023). To verify that the divergent phenotypes of TBCF10839 and its D8A6 mutant were caused by the disruption of *5PG21* gene and not by any polar effects or secondary mutations elsewhere in the TBCF10839 genome, we generated further mutants of TBCF10839 by targeting allelic replacement. A gentamicin resistance cassette was inserted within either *5PG21* gene at position 1,812 or eight nucleotides downstream of the stop codon.

**Figure 4:**
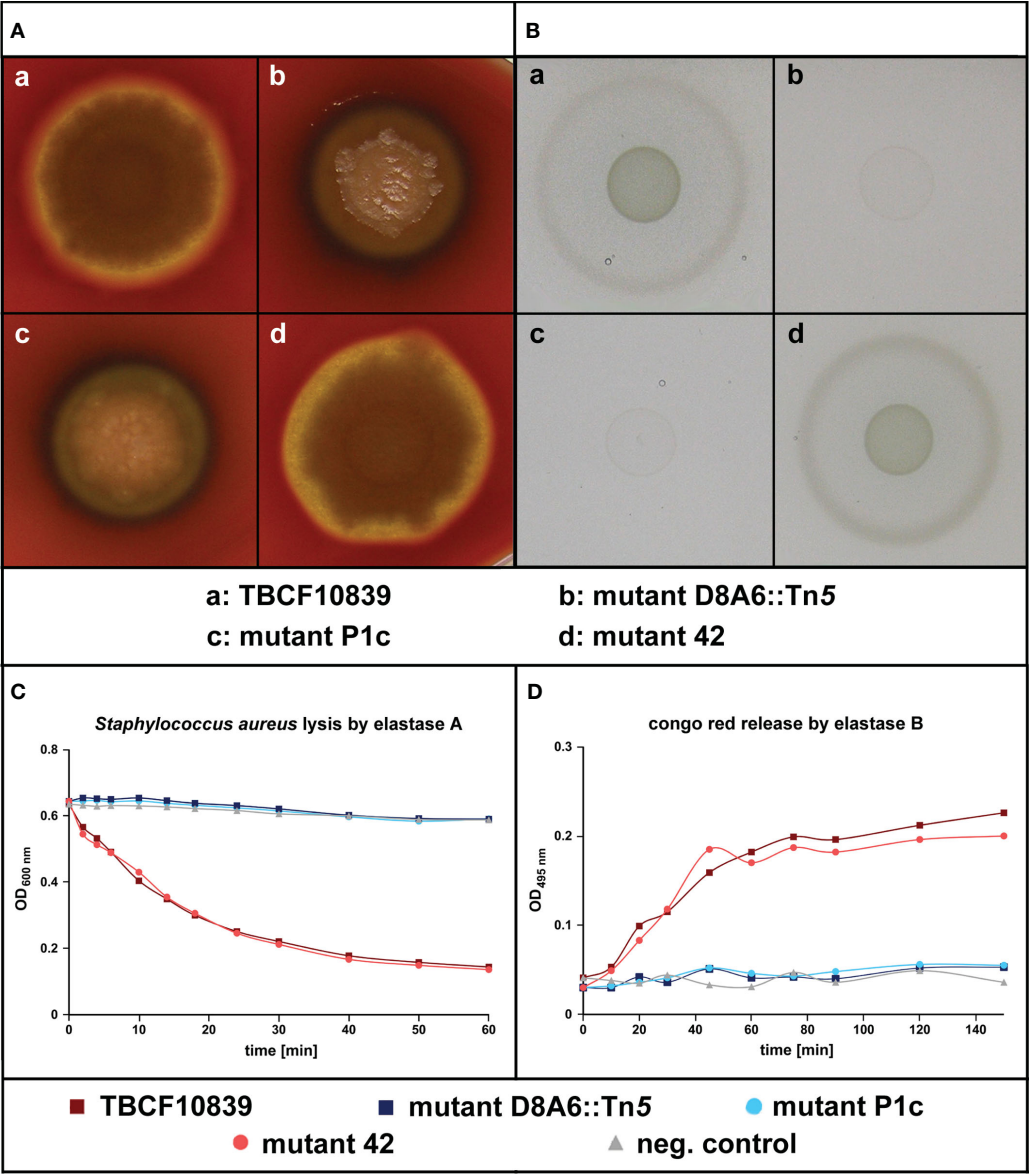
Test of quorum sensing-dependent phenotypes in *Pseudomonas aeruginosa* TBCF10839, its isogenic plasposon mutant D8A6 identified by a scan of the STM library, and the engineered isogenic mutants “P1c” and “42” that carry an insertion of the Gm gene cassette at position 1,812 of *5PG21* gene (“P1c”) or 8 bp downstream of the stop codon of *5PG21* (“42”). **(A)** Rhamnolipid-mediated hemolysis of bacterial colonies grown on blood agar. **(B)** Proteolytic digestion of casein of bacteria grown on M9–casein plates. **(C)** Spectrophotometry of the LasA-mediated lysis of *Staphylococcus aureus* cells by *P. aeruginosa* cell-free culture supernatant. **(D)** Spectrophotometry of the LasB-mediated release of Congo red from elastin impregnated with Congo red by *P. aeruginosa* cell-free culture supernatant. Wild-type *P. aeruginosa* TBCF10839 and its isogenic “42” insertion mutant were proficient in the secretion of rhamnolipid and protease. Conversely, the intragenic insertion mutants D8A6 and “P1c” were not hemolytic, degraded neither casein nor elastin, and did not cleave the pentaglycine bridges within the peptidoglycan network of the cell wall of *S. aureus.*

Randomly picked colonies of the intragenic mutant designated “P1” and of the 3′ mutant designated “42” were compared with TBCF10839 and D8A6 in their proficiency of quorum-sensing regulated phenotypes, i.e., hemolysis and protease secretion. Whereas TBCF10839 and the “42” mutant were hemolytic, the intragenic D8A6 and “P1” mutants did not lyse erythrocytes (**Figure 4A**). Likewise, TBCF10839 and the “42” mutant grew on casein as single carbon source (**Figure 4B**) and secreted LasA (**Figure 4C**) and LasB (**Figure 4D**), but all “P1” and D8A6 strains were deficient in protease secretion in the three bioassays. Since the extragenic “42” mutant showed wild-type behavior and the intragenic “P1” insertion mutant matched in phenotype with D8A6, we conclude that the loss of QS-regulated phenotypes was caused by the inactivation of *5PG21* and not by any polar effect.

### AHL and PQS production

**Figure 5:**
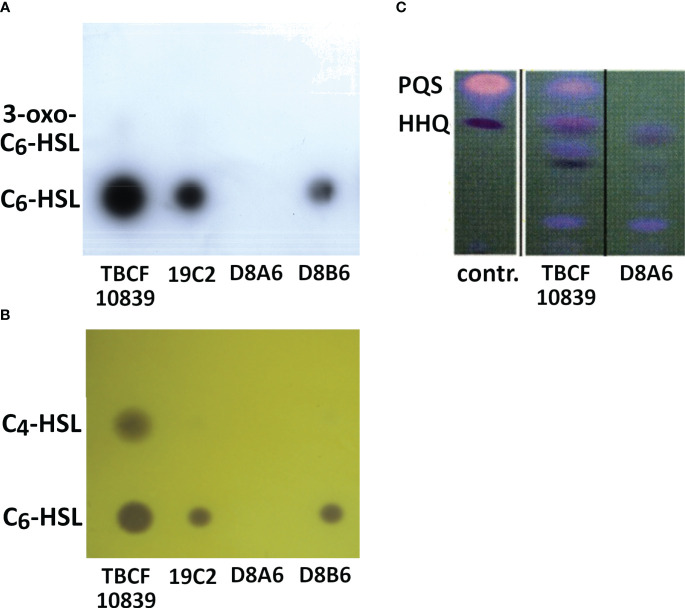
**(A, B)** Secretion of AHLs by *Pseudomonas aeruginosa* TBCF10839 and three isogenic plasposons of the STM library of TBCF10839 including D8A6 harboring the transposon insertion in *5PG21*. TLC of organic extracts of supernatants from bacteria grown for 15 h in ABC medium. **(A)** Analysis of secreted AHLs with the biosensor *Escherichia coli* MT102(pSB403), which is able to detect C_6_-HSL (lower dot) and 3-oxo-C_6_-HSL (faint upper dot, only visible for TBCF10839 and 19C2). **(B)** Analysis of secreted AHLs with the biosensor *Chromobacterium violaceum* CV026, which is able to detect C_6_-HSL (lower dot) and C_4_-HSL (upper dot). **(C)** Detection of quinolones by TLC of organic extracts of bacterial cultures grown for 15 h in LB. Right lane, TBCF10839 D8A6::Tn*5*; middle, TBCF10839; left, standards 3,4-dihydroxy-2-heptylquinoline (PQS) and 4-hydroxy-2-heptylquinoline (HHQ). No AHLs, PQS, or HHQ was detected in the D8A6 mutant. AHLs, *N*-acylhomoserine lactones; STM signature-tagged mutagenesis; TLC, thin-layer chromatography; LB, lysogeny broth.

The bioassays indicated that *5PG21* mutants were deficient in the QS-regulated secretion of rhamnolipid, elastase, and pyocyanine. Next, we wanted to explore to what extent the inactivation of *5PG21* affected the production of QS signal molecules: AHLs and 4-hydroxy-2-alkylquinoline (HAQs). As shown in **Figure 5**, the TBCF10839 strain was proficient in AHLs and 3,4-dihydroxy-2-heptylquinoline (PQS), but the D8A6 mutant produced only HAQ precursors, not PQS, the end product of the pathway. Moreover, no AHLs were detectable from D8A6 extracts on TLC indicator plates. In summary, D8A6 did not produce any QS signal molecules during growth in the ABC medium.

### Metabolite-mediated rescue of protease deficiency

The knock-out of the VirB4 homolog of the ICE-associated T4SS broadly attenuated the gene expression of numerous virulence determinants of TBCF10839 that to our knowledge are not orchestrated by a common master regulator. Hence, beyond transcriptional regulation, the phenotypes may also be modulated by other signals such as metabolites. To test this common sense hypothesis, we chose the simple model of the M9-casein plate whether supplementation with a metabolite could overcome the deficiency of *5PG21* knock-out mutants to metabolize casein.

Of 52 tested compounds, 35 metabolites within a concentration range of 1 to 50 mM promoted the digestion of casein by P1 and D8A6 bacteria (**Table 2**). The substrates and intermediates of the citric acid and the Entner–Doudoroff cycles and all amino acids except cysteine and methionine were capable of rescuing protease deficiency. Conversely, compounds such as peptides or hydrocarbons, which can be utilized by *P. aeruginosa* but do not belong to the core of the intermediary metabolism, often failed to induce bacterial growth and casein degradation (**Table 2**). In contrast to the P1 and D8A6 bacteria, the parental TBCF10839 strain and mutant 42 that carried an insertion downstream of *5PG21* could utilize all 52 tested compounds (**Table 2**). In summary, *5PG21* knock-out mutants P1 and D8A6 are compromised in their metabolic versatility to utilize nutrients.

**Table 2:** Growth of TBCF10839, its *5PG21* insertion mutants D8A6 and P1, and the 3′ insertion mutant 42 at 37°C on mineral medium agar plates supplemented with casein and metabolite.

Metabolite	TBCF10839	D8A6	P1	42
Amino acids				
Alanine	+	+	+	+
Arginine	+	+	+	+
Aspartate	+	+	+	+
Asparagine	+	+	+	+
Cysteine	+	ø	ø	+
Glutamate	+	+	+	+
Glutamine	+	+	+	+
Glycine	+	+	+	+
Histidine	+	+	+	+
Isoleucine	+	+	+	+
Leucine	+	+	+	+
Lysine	+	+	+	+
Methionine	+	ø	ø	+
Phenylalanine	+	+	+	+
Proline	+	+	+	+
Serine	+	+	+	+
Threonine	+	+	+	+
Tryptophan	+	+	+	+
Tyrosine	+	+	+	+
Valine	+	+	+	+
Organic acids				
Acetate	+	+	+	+
Benzoate	+	+	+	+
Butyrate	+	+	+	+
Citrate	+	+	+	+
Gluconate	+	+	+	+
Lactate	+	+	+	+
Malate	+	+	+	+
Pyruvate	+	+	+	+
Succinate	+	+	+	+
Alcohols				
Ethanol	+	+	+	+
Glycerol	+	+	+	+
Carbohydrates				
Galactose	+	+	+	+
Glucose	+	+	+	+
Maltose	+	+	+	+
Lipids				
Paraffin	+	+	+	+
Rapeseed oil	+	+	+	+
Urea cycle				
Ornithine	+	+	+	+
Argininosuccinate	+	ø	ø	+
Citrulline	+	ø	ø	+
Urea	+	ø	ø	+
Hydrocarbons				
Hexane	+	ø	ø	+
Heptane	+	ø	ø	+
Decane	+	ø	ø	+
Dodecane	+	ø	ø	+
Coenzymes				
AMP	+	ø	ø	+
ATP	+	ø	ø	+
NADH	+	ø	ø	+
Macromolecules				
albumin	+	ø	ø	+
casein	+	ø	ø	+
starch	+	ø	ø	+

+, growth; ø, no growth.

### Profile of metabolites

**Figure 6:**
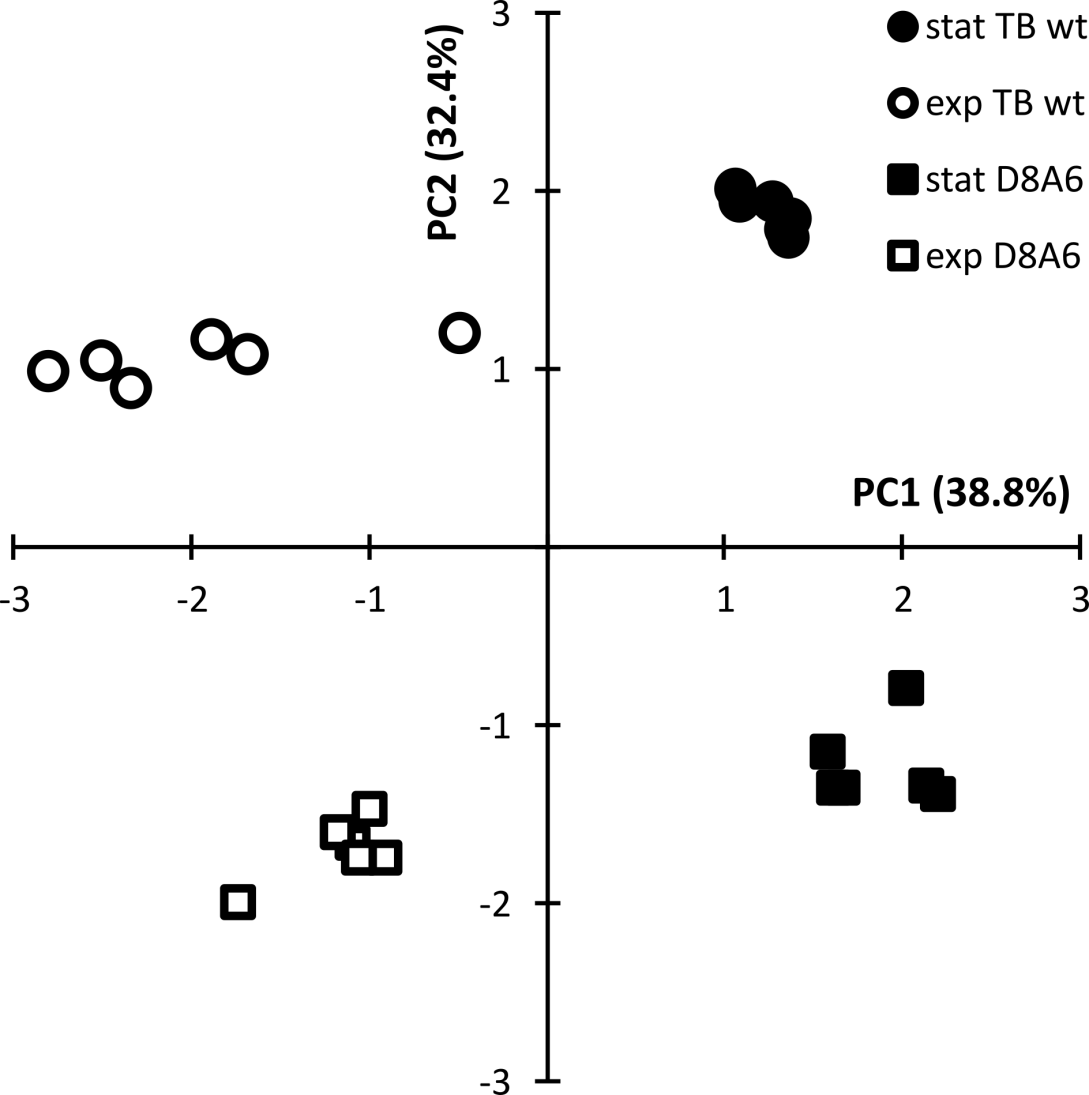
Principal component analysis of the quantitative spectrum of metabolites of *Pseudomonas aeruginosa* TBCF10839 (circle) and its D8A6 mutant (square) during fermentation in M9 medium with 40 mM of glucose at mid-exponential (open symbol) and early stationary phase (closed symbol).

**Figure 7:**
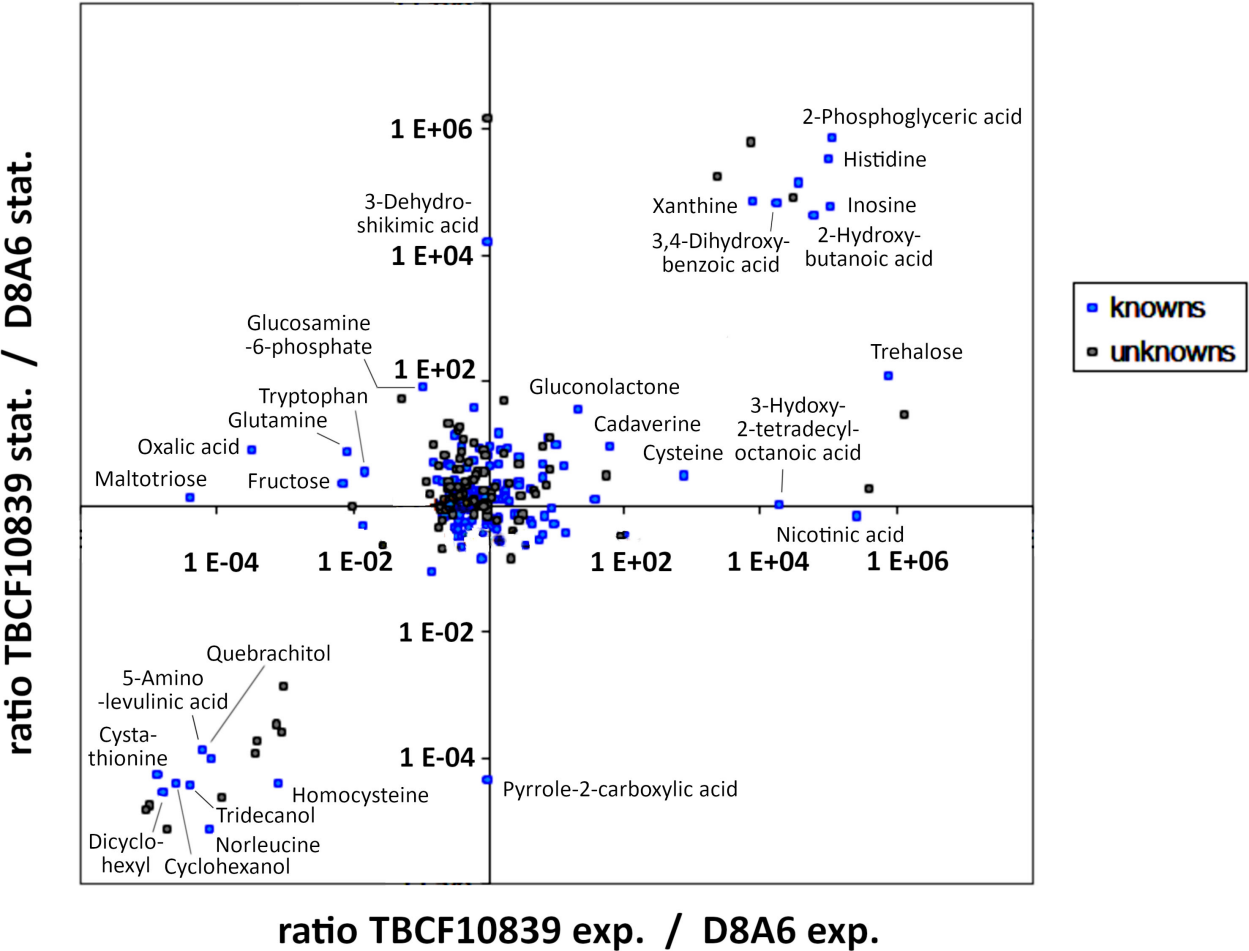
Intracellular metabolites of *Pseudomonas aeruginosa* TBCF10839 and its D8A6 mutant grown in M9 medium with 40 mM of glucose in a fermenter until mid-exponential and early stationary phase. The data points depict the ratio of the abundance of metabolites in TBCF10839 to that in the D8A6 plasposon mutant at a logarithmic scale.

The differential metabolic performance should also show up in a divergent profile of metabolites. *P. aeruginosa* is an aquatic organism that proficiently thrives in nutrient-poor habitats. Hence, we compared the spectrum of metabolites of wild-type TBCF10839 and D8A6 mutant during growth in a mineral medium with 40 mM of glucose as the sole carbon source. Principal component analysis separated the spectrum of metabolites by growth phase in the first dimension and by strain in the second dimension (**Figure 6**). Several compounds were more than 10,000-fold more abundant in either wild-type or mutant strain (**Figure 7**, **Table S3**). The wild-type strain exclusively produced trehalose that protects against abiotic stresses (Woodcock et al., 2021) and harbored intermediates of purine metabolism, serine biosynthesis, and aromatics degradation. The D8A6 plasposon mutant accumulated 5-aminolevulinic acid, the precursor of all tetrapyrrols, intermediates of methionine metabolism, several uncommon hydrocarbons, and quebrachitol, the methyl derivative of inositol known to be synthesized by plants but not by pseudomonads. As already shown in the plate assays, the mutant is apparently severely impaired in the utilization of nutrients and thus produces uncommon compounds that are not funneled into intermediary metabolism.

### Acute murine airway infection model

**Figure 8:**
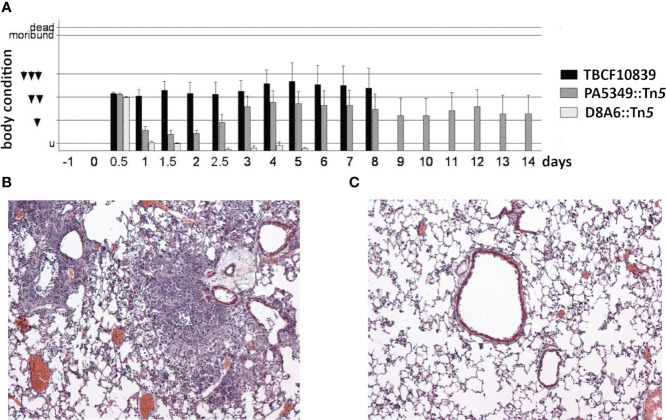
**(A)** Time course of the body condition score of groups of 10 C3H/HeN mice after intratracheal infection with 7.5 ×10^6^ CFU of *Pseudomonas aeruginosa* TBCF10839, its isogenic mutant D8A6, or another plasposon mutant (PA5349) identified in the STM scan. The applied dose was equivalent to LD50 for strain TBCF10839 but did not cause any death in D8A6-infected mice. The ethological body condition score represents the sum of nine individual scores (0, normal; 1, mildly affected; 2, severely affected) of vocalization, piloerection, posture, movement, breathing, activity, oculo-nasal secretion, grooming, and dehydration (untroubled, 0–1; mildly troubled, 2–4; moderately troubled, 5–7; substantially troubled, 8–10; moribund ≥ 11; death ≥ 16). Mice were moderately or substantially troubled during the whole observation period of 8 days if they had been infected with wild-type TBCF10839. Conversely, upon intratracheal infection with the isogenic TBCF10839 D8A6::Tn*5* mutant, the mice were acutely troubled at the 12-h time point, but by day 1 and thereafter, their behavior was normal during the whole observation period. **(B, C)** Pathohistology of the murine C3H/HeN lung on day 2 after intratracheal infection with TBCF10839 **(B)** or its isogenic mutant D8A6 **(C)**. **(B)** Strong peri- and intrabronchiolar infiltration of neutrophils and macrophages causes profound purulent pneumonia. **(C)** Only few leukocytes are seen around the bronchi with the same dose of the D8A6 mutant. Hematoxylin–eosin staining; original magnification, ×100. CFU, colony-forming unit; STM signature-tagged mutagenesis.

The D8A6 mutant was affected in quorum sensing and metabolic versatility. Quorum sensing regulates the social behavior and virulence of *P. aeruginosa* (Chadha et al., 2022). Hence, the pathogenicity of the highly virulent TBCF10839 should be attenuated in the AHL-deficient D8A6 mutant. Consistent with this hypothesis, intratracheal instillation of 7.5 × 10^6^ TBCF10839 caused 50% lethality in mice, whereas all mice survived the same dose of D8A6 bacteria (*p* = 0.016, Fisher’s exact test). Upon exposure to D8A6, the mice exhibited mild symptoms of disease 12 h after infection, but they recovered quickly and showed normal behavior all the time by day 1 and later (**Figure 8A**). Histology of their lungs by day 2 after infection displayed a minute infiltration of leukocytes, whereas infection with wild-type TBCF10839 caused severe purulent pneumonia (**Figures 8B, C**).

## Discussion

This showcase demonstrates that an element of the accessory genome can be essential for a *P. aeruginosa* strain to orchestrate its virulence determinants encoded in the core genome to generate modes of pathogenicity uncommon for this taxon. The index strain TBCF10839 can persist and replicate within neutrophils (Klockgether et al., 2013), the first defense line against almost all *P. aeruginosa* infections. Most *P. aeruginosa* strains can invade host cells and intracellularly diversify into vacuolar and cytosolic subpopulations that both contribute to pathogenesis (Kumar et al., 2022), but in addition to TBCF10839, the inactivation of neutrophils has been reported for only a few isolates, namely, strain CHA (Dacheux et al., 2000; Dacheux et al., 2001). *P. aeruginosa* survival inside cells has been shown to be modulated by the type III secretion system (T3SS) (Kroken et al., 2018), but in the case of strain TBCF10839, the transcriptome indicated that 5PG21 controls various virulence factors including QS, T6SS, and the biosynthesis of HCN and other antimetabolites.

Such a crucial role of a single gene of the accessory genome for the proper function of numerous, supposedly unrelated virulence functions, was *prima facie* unexpected. Mutant D8A6 of TBCF10839 defective in *5PG21* of the pKLC102 T4SS subtype was inconspicuous in its growth behavior *in vitro* in a minimal medium supplemented with various sole carbon sources. Conversely, D8A6 was avirulent *in vivo*. Since our allelic replacement mutants excluded any polar effect that may account for the *in vitro*, *ex vivo*, and *in vivo* phenotypes, we conclude that 5PG21 acts as a modulator of quorum sensing and virulence of the core genome independent of any other gene of PAGI5 of TBCF10839 including the upstream members of its nine-gene operon.

*5PG21* of TBCF10839 shares sequence identity with *5PG21* of strain PSE9 annotated as the VirB4 ATPase homolog of the “genomic island (GI)-type T4SS” (Kung et al., 2010). So far, elements of the GI-type T4SS have not been structurally characterized. Our current knowledge of the architecture of T4SS is based on studies on type A and type B systems encoded by the core genome of many Gram-negative and Gram-positive bacteria (Christie et al., 2005).

Bacterial type IV secretion systems are multiprotein nanomachines that translocate proteins or nucleic acids into target cells (Christie et al., 2014; Cabezón et al., 2015; Park et al., 2020). The structure of a few T4SS type A and type B systems has been visualized by electron microscopy (Christie et al., 2005; Low et al., 2014) or cryo-electron tomography (Hu et al., 2019; Park et al., 2020; Khara et al., 2021). A “minimized” T4SS (Khara et al., 2021) consists of an outer membrane core complex that spans the distal region of the periplasm and the outer membrane and an inner membrane complex that consists of four integral membrane components and two or three ATPases (VirB4 and VirD4 with or without VirB11) (Khara et al., 2021). VirB4 is the only ATPase that is present in all T4SS, and therefore, the presence of a VirB4-like protein constitutes a signature of a T4SS. VirB4 ATPases assemble into two side-by-side hexamers at the base of the secretion channel whereby the N-terminal domains are part of the inner membrane complex of the T4SS and the C-terminal domains extend into the cytoplasm (Khara et al., 2021).

Research on T4SS has focused on their role as effector or DNA translocation systems; for example, at least 15 genes of the ICE*clc* of *P. putida* including VirB4 are essential for ICE transfer in *P. putida* UWC1 (Daveri et al., 2023). Our case now shows a function of a VirB4 protein unrelated to conjugative transfer; i.e., the pKLC102-type VirB4 homolog of TBCF10839 behaved as a regulator of quorum sensing and virulence. Since we did not identify any plasposon mutant in the *clc*-type VirB4 homolog, we assume that the atypical features of 5PG21 VirB4 protein are transmitted by segments that exhibit no strong homology with the *clc* VirB4 sequence and are not embedded in the inner membrane complex. We are unaware of any other VirB4 protein that interferes with quorum sensing. Thus, we would like to ascribe the differential phenotypes between D8A6 and its TBCF10839 parent to “moonlighting functions” (Jeffery, 2018) of the pKLC102-type VirB4 that are not linked with its role within the inner membrane complex to promote translocation of molecules. *clc*-type islands spread across genus barriers by horizontal transfer (Klockgether et al., 2006; Johnson and Grossman, 2015; Delavat et al., 2017), but pKLC102 islands are confined to the species. Hence, considering the highly conserved sequence of *5PG21* orthologues in pKLC102-type genomic islands, further “moonlighting” 5PG21 VirB4 proteins is expected in a substantial portion of the *P. aeruginosa* population, but not in any other taxon.

pKLC102 is a highly mobile genomic island with spontaneous excision rates of up to 10^−1^, which are at least six orders of magnitude higher than those of *clc*-type islands in *P. aeruginosa* (Klockgether et al., 2007). Episomal copies replicate within the cell, and copy numbers have been estimated to be approximately 30 pKLC102 plasmids per host chromosome (Klockgether et al., 2007). Thus, albeit wild-type 5PG21 mRNA of TBCF10839 was not detectable in the Northern blotting, the inactivation of the multi-copy gene could influence the transcriptional program of the bacterium.

The D8A6 mutant was silent in QS and utilization of casein as a nutrient when grown under standard laboratory conditions of QS assessment. However, supplementation with metabolites of the core metabolism rescued QS and QS-regulated traits such as protease secretion. 5PG21 apparently represses quorum sensing when grown on complex and/or macromolecular nutrients that require activation of peripheral degradation pathways. In contrast, if the bacteria are fed with the easy-to-utilize substrates of intermediary metabolism, they become QS proficient. These findings suggest that 5PG21 may function as a metabolic sensor of virulence and quorum sensing. Future work should show whether these extra functions are also operating in other pKLC102-related ICEs that target tRNA^Lys^ genes in the *P. aeruginosa* chromosome.

## Data availability statement

The datasets presented in this study can be found in online repositories. The names of the repository/repositories and accession number(s) can be found below: https://www.ebi.ac.uk/ena, ERP001300 https://www.ncbi.nlm.nih.gov/, PRJNA975170.

## Ethics statement

The studies involving human participants were reviewed and approved by Ethics Committee of Hannover Medical School, study no. 6790. The patients/participants provided their written informed consent to participate in this study. The animal study was reviewed and approved by Niedersächsisches Landesamt für Verbraucherschutz und Lebensmittelsicherheit/LAVES; approval number: 04/787.

## Author contributions

LW and BT conceived the study. LW constructed and screened the STM library. JK performed genome sequencing and bioinformatic analyses. JK and A-SH generated mutants by allele replacement. LW, PS, SH, JFP, and BT worked on transcriptome and metabolome and analyzed the datasets. LW, JK, and SH performed quorum sensing-related bioassays and studied bacterial phenotypes on plates. AM performed animal infection experiments. EG, LE, and BT provided funding and resources. LW, JK, and BT wrote the paper. All authors contributed to the article and approved the submitted version.

## Supplementary material

Supplementary Table 1 Gene chip comparison of the gene expression of TBCF10839 and the D8A6 mutant grown aerobically in ABC minimal medium: Significantly higher mRNA transcript levels in the D8A6 mutant.

Supplementary Table 2 Gene chip comparison of the gene expression of TBCF10839 and the D8A6 mutant grown aerobically in ABC minimal medium: Significantly higher mRNA transcript levels in TBCF10839.

Supplementary Table 3 Fermentation of wild type TBCF10839 and D8A6 mutant in mineral medium with 40 mM glucose: Metabolites identified by GLC/MS in the mid-exponential and early stationary growth phase.
